# Novel mutations in actionable breast cancer genes by targeted sequencing in an ethnically homogenous cohort

**DOI:** 10.1186/s12881-019-0881-0

**Published:** 2019-09-02

**Authors:** Hosneara Akter, Nasima Sultana, Nazrana Martuza, Aaysha Siddiqua, Nushrat Jahan Dity, Md. Atikur Rahaman, Bisan Samara, Ahmed Sayeed, Mohammed Basiruzzaman, Mohammad Mizanur Rahman, Md. Rashidul Hoq, Md. Robed Amin, Md. Abdul Baqui, Marc Woodbury-Smith, K. M. Furkan Uddin, Syed S. Islam, Rayhana Awwal, Bakhrom K. Berdiev, Mohammed Uddin

**Affiliations:** 1 NeuroGen Technologies Ltd., Genetics and Genomic Medicine Centre, Dhaka, Bangladesh; 2 Directorate General of Health Services, Ministry of Health and Family Welfare, Dhaka, Bangladesh; 3 Mohammed Bin Rashid University of Medicine and Health Sciences, College of Medicine, Dubai Healthcare City, Building 14, Dubai, United Arab Emirates; 4 Holy Family Red Crescent Medical College, Dhaka, Bangladesh; 5 Department of Pediatrics, Bangabandhu Sheikh Mujib Medical University (BSMMU), Dhaka, Bangladesh; 6 Department of Medicine, Dhaka Medical College, Dhaka, Bangladesh; 7 Institute of Neuroscience, Newcastle University, Newcastle upon Tyne, UK; 8 The Centre for Applied Genomics, The Hospital for Sick Children, Toronto, Ontario Canada; 9 Molecular Oncology, King Faisal Specialist Hospital and Research Centre, Riyadh, Saudi Arabia; 10 Sheikh Hasina National Institute of Burn & Plastic Surgery, Dhaka, Bangladesh

**Keywords:** Breast Cancer, Pathogenic, VUS, *BRCA1*, *BRCA2*

## Abstract

**Background:**

Genetic testing is becoming an essential tool for breast cancer (BC) diagnosis and treatment pathway, and particularly important for early detection and cancer prevention. The purpose of this study was to explore the diagnostic yield of targeted sequencing of the high priority BC genes.

**Methods:**

We have utilized a cost-effective targeted sequencing approach of high priority actionable BC genes (*BRCA1*, *BRCA2*, *ERBB2 and TP53)* in a homogeneous patient cohort from Bangladesh (*n* = 52) by using tumor and blood samples.

**Results:**

Blood derived targeted sequencing revealed 25.58% (11/43) clinically relevant mutations (both pathogenic and variants of uncertain significance (VUS)), with 13.95% (6/43) of samples carrying a pathogenic mutations. We have identified and validated five novel pathogenic germline mutations in this cohort, comprising of two frameshift deletions in *BRCA2,* and missense mutations in *BRCA1*, *BRCA2* and *ERBB2* gene respectively. Furthermore, we have identified three pathogenic mutations and a VUS within three tumor samples, including a sample carrying pathogenic mutations impacting both *TP53* (c.322dupG; a novel frameshift insertion) and *BRCA1* genes (c.116G > A). 22% of tissue samples had a clinically relevant *TP53* mutation. Although the cohort is small, we have found pathogenic mutations to be enriched in *BRCA2* (9.30%, 4/43) compare to *BRCA1* (4.65%, 2/43). The frequency of germline VUS mutations found to be similar in both *BRCA1* (4.65%; 2/43) and *BRCA2* (4.65%; 2/43) compared to *ERBB2* (2.32%; 1/43).

**Conclusions:**

This is the first genetic study of BC predisposition genes in this population, implies that genetic screening through targeted sequencing can detect clinically significant and actionable BC-relevant mutations.

**Electronic supplementary material:**

The online version of this article (10.1186/s12881-019-0881-0) contains supplementary material, which is available to authorized users.

## Background

Breast cancer (BC) is the most common type of cancer among women, impacting 2 million new cases and causes over 600,000 deaths worldwide [1]. While the prevalence of BC is increasing globally, it is critical to screen known BC genes to improve breast cancer survival and early detection, especially in developing countries where majority of women with BC are diagnosed at an advanced stage. The five-year survival rate is below 40% in low-income countries, 60% in middle-income countries and 80% or over in North America, Sweden and Japan where early detection and different treatment options are available [2].

Traditionally, breast self-examination, clinical breast assessment and mammography all have been used alone or in combination to screen BC and facilitate early detection of potentially malignant breast lesions. In recent years, genetic screening has become a critical tool for BC assessment, diagnosis and in guiding treatment choices [3, 4].Mutation profiling for BC has been an integral part of clinical care since the discovery of the *BRCA1* and *BRCA2* genes [5–7].Cases with *BRCA1* and *BRCA2* pathogenic mutations have a significantly increased risk of developing BC before the age of 50 years [8–11]. Breast cancer risk for late onset cases (above 70 years old) who carry pathogenic mutation in *BRCA1* and *BRCA2* is 57 and 50%, respectively [12]. These numbers are higher than any other studied mutations associated with hereditary (familial) BC. Importantly, the frequency distribution of some of the *BRCA* mutations varies by population studied, suggesting a population specific mutational profile.

Another important gene is *TP53* that was originally identified as a risk factor for Li–Fraumeni syndrome. *TP53* mutations are the most frequent genetic abnormalities in BC tumors. Approximately 30% of all BC tumors reported to have a mutation in *TP53* and mutation within this gene is also associated with poor prognosis [13]. The mutation frequency varies depending on the tumor subtypes, with mutations in 26% luminal, 50% in HER2 amplified tumors, and 88% in basal-like subtypes [13].

It has been reported that the*TP53* mutation status may influence the patient’s response to treatment, and determine resistance to several chemotherapy drugs [14–16]. Although genetic mutation screening is becoming an essential test for BC diagnosis and therapeutics, the cost associated with whole genome sequencing is still high and not a feasible option for clinical practice in developing countries. Given these challenges, we explored the diagnostic yield of targeted sequencing of the high priority BC genes *BRCA1*, *BRCA2*, *TP53* and *ERBB2*.

## Methods

### Study subjects

The cohort comprised of 52 individuals (43 blood and 9 breast tissue samples) with age ranges between 30 and 70 years, including 30.77% (16/52, 13 blood and 3 tissue samples) between 30 and 39 years, 36.54% (19/52, 16 blood and 3 tissue samples) between 40 and 49 years and 32.69% (17/52, 14 blood and 3 tissue samples) between 50 and 70 years (Table 3). Of the 52, 76.92% (40/52, 38 blood and 2 tissue samples) had a positive family history of breast cancer and 61.53% (32/52, 23 blood and 9 tissue samples) were diagnosed with breast cancer. 30.77% (16/52, all are blood samples) had symptoms of breast lump, pain and swelling but were, as yet, undiagnosed. Although the rest 7.70% (4/52) had no symptoms, these individuals were included into the cohort due to positive family history. Of the 32 diagnosed breast cancer patients, 15.62% (5/32, all are blood samples) were in stage I, 53.13% (17/32, 12 blood and 5 tissue samples) were in stage II, 25% (8/32, 6 blood and 2 tissue samples) were in stage III and 6.25% (2/32, all are tissue samples) did not provide stage information. These cases went through clinical assessment for breast cancer at multiple centers between January, 2017 to August, 2018 in Dhaka, Bangladesh. They were prospectively recruited from Dhaka Medical College and Hospital, Holy Family Red Crescent Medical College and Hospital, Oncology Center and General Hospital, and IbnSina Diagnostic and Imaging Center of Bangladesh. The study was approved by the Institutional Review Board of Holy Family Red Crescent Medical College, and all samples were collected with written informed consent.

### DNA extraction and amplification

DNA was extracted from breast tissue and blood sample using GeneJET Genomic DNA Purification Kit (Thermo Fisher Scientific, USA) and ReliaPrep™ Blood gDNA isolation kit (Promega, USA) respectively according to manufacturer protocols. The concentration and quality of DNA was measured using NanoPhotometer C40 (Implen, Germany).We have developed a panel comprised of four high impact genes in breast cancer using high throughput sequencing technology. We have designed (using Primer 3 plus software, IDT and UCSC Genome Browser) 52 sets of primers targeting all exons and splicing junctions of *BRCA1*, *BRCA2*, *TP53* and *ERBB2* genes (Additional file 1). In total, 13 sets of multiplex PCR were carried out to amplify all the 52 amplicons (Additional file 1: Table S1-S5) using GoTaq® Hot Start Colorless Master Mix and GoTaq *Long PCR Master Mix* (Promega, USA). The amplicons were visually confirmed by 0.8% agarose gel electrophoresis. After confirmation, amplicons were purified using the Agencourt AMPure XP PCR purification bead (Beckman Coulter, Pasadena, CA) and quantified using the QuantiFluor® ONE dsDNA System (Promega, USA). Then 1 ng target amplicons were used for library preparation. Nextera XT library preparation kit (Illumina, Inc., San Diego, CA), using the manufacturer’s recommended protocol, was used for library preparation.

### DNA sequencing and analysis

The barcoded libraries were subsequently sequenced at NeuroGen Technologies Ltd. genetics and genomics laboratory using the MiniSeq sequencer (Illumina, Inc. USA) with miniseq mid output kit, which generated 150 base paired-end sequence reads. The run was set up in local run manager that is an integrated computer software platform of MiniSeq, which uses a Burrows-Wheeler Aligner (BWA) [17] and the Genome Analysis ToolKit (GATK 4.0.11.0) [18] for converting raw sequence reads to Binary Alignment/Map (BAM) and Variant Call Format (VCF) v4.1 files. Sequencing proceeded on the platform for 24 h with default settings within the MiniSeq system. For quality control, a Q-score of 30 was used as a threshold for each identified variant, corresponding to a 1:1000 error rate. The average coverage is 91.42% for all four genes (for 52 primer sets) and 96% of the amplicons have a mean sequencing coverage of 50X.We have used ANNOVAR (2018Apr16 version) for functional annotation of the variants. For genomic annotations, we have also used GenomeArc, a custom genetic annotation tool. Mutation classification analysis was conducted based on American College of Medical Genetics (ACMG) guidelines [19]. Sanger sequencing was used to confirm the novel mutations [20].

### Sanger sequencing

All novel variants identified using MiniSeq (Illumina, Inc. USA) were validated by Sanger sequencing. For this purpose, PCR was performed using genomic DNA as a template and primer pairs flanking the deleterious variant sites. We have designed primers (Additional file 1: Table S6) for this validation using Primer 3 plus software, IDT and UCSC Genome Browser. The PCR products were visually confirmed by 2.0% agarose gel electrophoresis. The products were then purified using the Wizard® SV Gel and PCR Clean-Up System (Promega, USA) according to the manufacturer’s instructions. Cycle sequencing was performed using purified PCR products as template and BigDye® Terminator v3.1 (Applied Biosystem, USA). Then sequencing was performed using 3500 DNA Analyzer (Applied Biosystem, USA). Subsequently Sanger sequencing data were analyzed using Sequence Scanner v2.0 (Applied Biosystem, USA).

## Results

We have used nine tumor samples to sequence four bonafide BC genes and identified three pathogenic mutations and a VUS within three tumor samples (Table 1, Table 3 and Additional file 1: Table S7). The first patient (Table 1), carries a *TP53* (c.733 G > A) missense pathogenic mutation in exon 7 that impacts the DNA binding domain of *TP53* protein. The second patient (Table 1) carries 2 pathogenic mutations in both *BRCA1* (c.116 G > A) and *TP53* (c.322dupG) gene. A variant of uncertain significance (VUS) in *BRCA2* (c. 2459A > G) was identified in the third patient (Table 1).

**Table 1:** List of clinically relevant mutations within the *BRCA1*, *BRCA2* and *TP53* genes in resected breast tumor tissues in our Bangladeshi cohort

Sample Id	Gene Name	Coordinate [position] (hg19)	Mutation Types	Significance	Exon	Nucleotide change	Amino acid change
1	*TP53*	7,577,548	Nonsynonymous	pathogenic	7	c.733G > A	p.Gly245Ser
2	*TP53*	7,578,490	frameshift insertion*	pathogenic	5	c.322dupG	p.Val108Glyfs*
	*BRCA1*	41,267,761	Nonsynonymous	pathogenic	3	c.116G > A	p.Cys39Tyr
3	*BRCA2*	32,910,951	Nonsynonymous	VUS	11	c.2459A > G	p.Asp820Gly

*defines the novel variants in our cohort

**Fig. 1:**
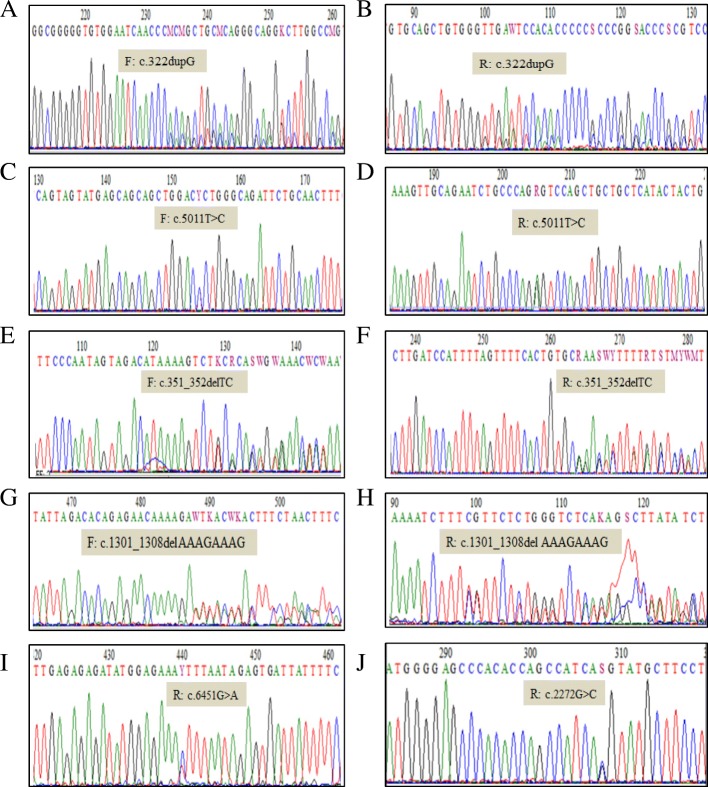
Sequence chromatograms of all novel mutations detected in BRCA1, BRCA2, TP53, and ERBB2 genes. **a** and **b** Forward and reverse strands sequence of pathogenic frameshift insertion mutation c.322dupG in TP53 gene. **c** and (**d**) Forward and reverse strands sequence of pathogenic missense mutation c.5011 T > C in BRCA1 gene. **e** and **f** Forward and reverse strands sequence of pathogenic frameshift deletion mutations c.351_352delTC in BRCA2 gene. **g** and **h** Forward and reverse strands sequence of pathogenic frameshift deletion mutationc.1301_1308del AAAGAAAG in BRCA2 gene. **i** and **j** Reverse strand sequence of missense mutation c.6451G > A and c.2272G > Cin BRCA2 and ERBB2 genes respectively

The overall clinical yield from the 43 blood specimens was 11 clinically relevant (pathogenic or VUS) mutations (25.58%), including 13.95% (6/43) patients with clinically pathogenic mutations (Table 2, Table 3 and Additional file 1: Table S7). Among these 6 variants, 4 are novel pathogenic germline mutations (Table 2) comprising 2 frameshift deletions in *BRCA2* (Table 2), 1 missense mutation impacting *BRCA2* (Table 2) and 1 missense mutation impacting *BRCA1* (Table 2) gene*.* Two novel frameshift sequence variants are c.1301_1308delAAAGAAAG in exon 10 and c.351_352delTC in exon 4 of *BRCA2*. These mutations truncated the protein at amino acid positions of 118 and 436. In *BRCA2* gene, another novel missense variant c.6451 G > A, was found in exon 11. Another two novel mutations c.5011 T > C and c.2272 G > C were found in *BRCA1* and *ERBB2,* respectively (Table 2, Table 3 and Additional file 1: Table S7). All the novel mutations (c.322dupG, c.351_352delTC, c.1301_1308delAAAGAAAG, c.5011 T > C, c.6451 G > A and c.2272 G > C) were further validated by Sanger sequencing (Fig. 1).

**Table 2:** List of clinically relevant variants detected within the *BRCA1, BRCA2* and *ERBB2* genes applying targeted sequencing from blood derived DNA samples in our breast cancer cohort recruited from Bangladesh

Sample Id	Gene name	Coordinate [position] (hg19)	Mutation Types	Significance	Exon	Nucleotide change	Amino acid change
1	*BRCA2*	32,931,983	Stopgain	pathogenic	16	c.7722 G > A	p.Trp2574Ter
2	*BRCA2*	32,906,729	Nonsynonymous	VUS	10	c.1114 A > C	p.Asn372His
3	*BRCA2*	32,907,407	Nonsynonymous	VUS	10	c.1792A > G	p.Thr598Ala
4	*BRCA1*	41,222,983	Nonsynonymous^*^	Pathogenic	16	c.5011 T > C	p.Met1671Val
5	*BRCA1*	41,245,262	Nonsynonymous	VUS	10	c.2286A > T	p.Arg762Ser
6	*BRCA1*	41,243,553	Nonsynonymous	VUS	10	c.3995 C > A	p.Gly1332Val
7	*BRCA2*	32,906,916–32,906,923	frameshift deletion ^*^	Pathogenic	10	c.1301_1308del AAAGAAAG	p.Lys436Phefs*
8	*BRCA1*	41,246,489	Stopgain	pathogenic	10	c.1058G > A	p.Trp353Ter
9	*BRCA2*	32,899,247	frameshift deletion ^*^	Pathogenic	4	c.351_352delTC	p.Arg118Hisfs*
10	*ERBB2*	37,880,988	Nonsynonymous^*^	VUS	24	c.2272 G > C	p.Val758Leu
11	*BRCA2*	32,914,943	Nonsynonymous^*^	Pathogenic	11	c.6451 G > A	p.Val2151Ile

*defines the novel variants in our cohort

**Table 3:** Descriptive statistics of the breast cancer cohort

Description	History of study cases (specimen type: blood)	%	History of study cases (specimen type: tissue)	%
Number of samples	43/52	82.69	9/52	17.31
Age range (years)				
30–39	13/43	30.23	3/9	33.33
40–49	16/43	37.21	3/9	33.33
50–70	14/43	32.56	3/9	33.33
Clinical information				
Breast Cancer	23/43	53.49	9/9	100.00
Stage I	5/23	21.74	0/9	0.00
Stage II	12/23	52.17	5/9	55.55
Stage III	6/23	26.09	2/9	22.22
Stage unknown	N/A	N/A	2/9	22.22
Grade I	3/23	13.04	–	–
Grade II	13/23	56.52	–	–
Grade III	6/23	26.09	–	–
Grade unknown	1/23	4.35	9/9	100
Breast lump	11/43	25.58	–	–
Breast pain and secretion	5/43	11.63	–	–
No symptoms	4/43	9.30	–	–
Family History				
Positive	38/43	88.37	2/9	22.22
Negative	5/43	11.63	3/9	33.33
Unknown	–	–	4/9	44.44
Mutation found(including VUS and Pathogenic)	11/43	25.58	3/9 (1 patient carried 2 mutations. So total number of mutations in 3 patients are 4)	33.33
*BRCA1*(V + P)	4/43	9.30	1/9	33.33
*BRCA2*(V + P)	6/43	13.95	1/9	33.33
*TP53*(P)	–	–	2/9	22.22
*ERBB2*(V)	1/43	2.32	–	–

Note: V:VUS and P: Pathogenic

In this cohort, we have also identified 6 known clinically relevant variants that include 2 known pathogenic variants, c.7722 G > A in exon 16 of *BRCA2* and c.1058 G > A in exon 10 of *BRCA1* (Table 2) and the remaining 4 mutations are missense. Out of 4, 2 are located in exon 10 of *BRCA2* gene and 2 are located in exon 10 of *BRCA1* gene (Table 2).

## Discussion

Although large scale targeted sequencing has identified new BC mutations in the developed countries [21], data on the mutational architecture in low and middle income countries, such as Bangladesh, remains limited [22]. The recent increase of BC prevalence and the detection of bonafide causal genes imply the exploration of the genomic landscape of BC in countries such as Bangladesh to facilitate early diagnosis and screening to target treatments appropriately. We have designed a targeted gene-sequencing panel for known high-risk breast cancer genes, namely *BRCA1*, *BRCA2*, *TP53* and *ERBB2*, which was after proper quality control incorporated into the diagnostic pathway.

The incidence and prevalence of BC in Bangladesh is mostly unknown due to the lack of population-based cancer registries either locally or centrally. In Bangladesh, most patients are diagnosed at an advanced stage of the disease and suffer from worse treatment outcome due to lack of breast cancer awareness, inadequate access to healthcare and excessive treatment related cost. Genetic screening for *BRCA1/2* and *TP53* genes and identification of novel mutation and variants serves as key roles for timely diagnosis, treatment, counseling, follow-up of patients and management of disease [23]. *BRCA1/2* carriers now can have targeted therapies that apply parp-inhibitor to facilitate DNA repair process in tumor cell. It was found that the oral PARP inhibitor olaparib has antitumour activity in those patients who have lost *BRCA1* or *BRCA2*-associated DNA repair [24]. Similarly, for *TP53* mutations multiple targeted therapies shown promising result to improve the survival rate for *TP53* mutation carriers [25].

We have identified 25.58% (11/43) germline (blood derived DNA) clinically relevant mutations with familial cancer history. Results obtained in our cohort corroborate the previously reported studies that investigated only BRCA genes within familial patients [26]. Of the 11 germline mutations, we identified 10 sequence variants in *BRCA1* and *BRCA2* genes, including 13.95% (6/43) cases carrying a pathogenic mutation and the frequency correlate strongly with Cyprus study that reported a similar clinical yield of 13% for BRCA genes [27]. Despite of small cohort size, we have found germline pathogenic mutation impact on *BRCA2* (9.30%, 4/43) which is approximately 2-fold higher than *BRCA1* (4.65%, 2/43), suggesting that *BRCA2* is frequently mutated or altered in our cohort. This is also consistent with a Chinese cohort where *BRCA2* was shown to have a higher prevalence  compared to *BRCA1* [28]. The frequency of germline VUS mutations are same in both *BRCA1* (4.65%; 2/43) and *BRCA2* (4.65%; 2/43) compared to *ERBB2* (2.32%; 1/43).

Of the 11germline mutations, we have found 2 novel pathogenic frameshift deletions (Table 2) in exon 10 and 4 of *BRCA2* (Table 2) gene respectively. These mutations truncated the BRCA2 protein at amino acid positions of 118 and 436. The truncated protein lacks BRCA2 functional domains (RAD51 and a DNA binding domain) that plays an important role in the homologous recombination (HR) repair of damaged DNA in cells [29, 30]. In the exon 10 of *BRCA2* gene we have also identified 2 known missense mutations (Table 2). Among these two, one is p.Asn372His (rs144848) which is a common non-synonymous polymorphism in exon 10 of the *BRCA2* gene [31]. The change from A to C in the rs144848 polymorphism results in an asparagine-to-histidine transition (p.N372H) which may affect *BRCA2* structure at residues 290–453, a region responsible to interact with the histone acetyl transferase P/CAF prior to transcriptional activation of target genes [32]. Multiple independent studies have identified varying degree of association of rs144848 p.N372H polymorphism in cancer risk, but the susceptibility to breast cancer is still inconclusive [33–38]. Another variant (p.Thr598Ala) within exon 10 was found to have conflicting pathogenicity within literature [39]. We have identified another novel missense variant p.Val2151Ile (Table 2) in exon 11 of *BRCA2* gene. We have also identified a known pathogenic mutation p.Trp2574Ter (Table 2) in the exon 16 of *BRCA2* gene. This variant likely to cause the protein to be abnormally truncated at the amino acid position 2574. It is located within the DNA-binding domain (DBD: belongs to 2481–3186 amino acids) of *BRCA2* gene that binds single-stranded DNA (ssDNA) and double-stranded DNA (dsDNA). The DBD contains five components: a 190-amino-acid α-helical domain, three oligonucleotide binding (OB) folds that are ssDNA-binding modules, and a tower domain (TD) that protrudes from OB2 and binds dsDNA [40]. The helical domains, OB1 and OB2 also associate with deleted in split-hand/split-foot syndrome (DSS1), which has been linked to *BRCA2* protein stabilization [40–43]. This variant is pathogenic and was found in the patients of Hereditary Breast and Ovarian Cancer syndrome [39].

Out of 10 sequence variants in *BRCA1* and *BRCA2* gene, we have found 1 novel missense mutation p.Met1671Val (Table 2) in exon 16 of *BRCA1* gene. This mutation is located within the phosphoprotein-binding C-terminal BRCT domain which is critical for the tumor suppression function of *BRCA1* gene [44]. We have also identified 3 known mutations in the exon 10 of *BRCA1* gene. Among these mutations, 2 are missense mutations p.Arg762Ser and p.Gly1332Val (Table 2) and another 1 is stopgain mutation p.Trp353Ter (Table 2). The missense mutations were previously identified in patients with breast cancer and hereditary cancer-predisposing syndrome and were defined as VUS [39, 45]. The stopgain mutation was previously identified in the patients of hereditary breast and ovarian cancer syndrome and was defined as pathogenic [39, 45, 46].

We have also identified a novel missense mutation p.Val758Leu (Table 2) in the exon 24 of *ERBB2* gene. *ERBB2* kinase domain mutation occurs in human cancers such as gastric, breast, and colorectal cancers, and suggested that alterations of ERBB2-mediated signaling pathway by *ERBB2* mutations alone or together with K-RAS mutations may contribute to the development of human cancers [47].

We also identified four mutations in 3 of the 9 breast tumor tissue samples, including 22% (2/9) with pathogenic mutations in *TP53* gene, comparable to data from the International Agency for Research on Cancer (IARC) [48]. The patient carrying this frameshift mutation also has another pathogenic mutation, c.116 G > A in the exon 3 of *BRCA1* gene which was previously identified in the patients of hereditary breast and ovarian cancer syndrome, neoplasm of the breast and hereditary cancer-predisposing syndrome [39, 49]. The majority of the mutations occur in the hot-spots exons 5, 7 and 8 [50, 51] of *TP53* gene. *Mazars* et al found that among patients with ovarian cancer, all p53 mutations clustered in exons 5 and 7 [52]. Somatic *TP53* gene alterations are frequent in most human cancers, ranging from 5 to 80% depending on the type, stage, and etiology of tumors [53]. BC tumors also are impacted by frequent *TP53* mutations and based on the mutation type and location, cancer subtype can be classified based on treatment and prognosis. Hence, *TP53* mutated warrant targeted treatment depending on the *TP53* mutation status [13]. Inherited *TP53* mutations predispose to a wide spectrum of early-onset cancers and are associated with Li-Fraumeni and Li-Fraumeni-like syndrome (LFS and LFL), respectively [54]. We also identified another missense mutation p.Asp820Gly (Table 1) in the exon 11 of *BRCA2* gene that was previously identified in the patients of familial breast-ovarian cancer syndrome [39, 55].

## Conclusion

In this study, we have designed a cost effective targeted gene panel to investigate the mutational landscape of 4 high impact breast cancer genes (*BRCA1, BRCA2, ERBB2* and *TP53*) in breast tumor and in blood. This is the first paper on breast cancer mutation screening on Bangladeshi population that is ethnically a homogenous population. We have identified 5 novel mutations that are extremely rare in other populations, as well as known pathogenic mutations for breast cancer. The accumulation of more genomic data will be able to quantitate their true prevalence, as well as their association with other disease phenotypes. We have also observed clinically relevant mutation enrichment within *BRCA2* genes. Our list of detected variants of unknown significance will also provide the opportunity to conduct further research to clarify ambiguous pathogenicity. In recent years, breast cancer becoming a major issue in developing countries due to low survival rate compared to developed countries. The identification of novel mutations from a homogenous population will add great value to breast cancer genetics. Moreover, this paper will bring broader community awareness on cancer genetic tests and the implementation of precision medicine in general for the country.

## Additional file

Additional file 1: Supplementary Information. **Table S1.** Detail of BRCA1 primers used. **Table S2.** Detail of BRCA2 primers used. **Table S3.** Detail of ERBB2 primers used. **Table S4.** Detail of TP53 primers used. **Table S5.** Detail of multiplex PCR. **Table S6.** Detail of primers for Sanger sequencing. **Table S7.** Detail of the history of study cases who have mutations. (DOCX 41 kb)

## Data Availability

The datasets used and/or analyzed during the current study are available from the corresponding author on reasonable request. Confidential patient data are not shared.

## Abbreviations

ACMG: American College of Medical Genetics
BAM: Binary Alignment/Map
BC: Breast Cancer
BWA: Burrows-Wheeler Aligner
DBD: DNA-Binding Domain
dsDNA: double-stranded DNA
DSS1: Deleted in Split-hand/Split-foot Syndrome
GATK: Genome Analysis ToolKit
HR: Homologous Recombination
IARC: International Agency for Research on Cancer
IERC: Institutional Ethical Review Committee
OB: Oligonucleotide Binding
ssDNA: single-stranded DNA
TD: Tower Domain
VCF: Variant Call Format
VUS: Variant of Uncertain Significance
