# Supplementary material for: Novel mutations in actionable breast cancer genes by targeted sequencing in an ethnically homogenous cohort
Source: BMC Med Genet. 2019 Sep 2;20:150. doi: 10.1186/s12881-019-0881-0 (PMC6721087; doi:10.1186/s12881-019-0881-0)
Supplement: Supplementary file 1 — Supplementary Information. Table S1. Detail of BRCA1 primers used. Table S2. Detail of BRCA2 primers used. Table S3. Detail of ERBB2 primers used. Table S4. Detail of TP53 primers used. Table S5. Detail of multiplex PCR. Table S6. Detail of primers for Sanger sequencing. Table S7. Detail of the history of study cases who have mutations. (DOCX 41 kb) [file 12881_2019_881_MOESM1_ESM.docx]

**Supplementary Information: Novel Mutations in High Priority Actionable Breast Cancer Genes by Targeted Sequencing in an Ethnically Homogenous Cohort**

For our target genes *BRCA1, BRCA2, ERBB2/HER2*, and *TP53* we have used UCSC Genome browser, Primer 3plus and IDT softwares.

**Selecting Target Regions (UCSC Genome Browser)**

At first the target gene was opened at UCSC genome browser. Then sequentially the exonic region selected by “drag and select” method. For example, at 1st target Exon 1 of BRCA1, drag the cursor to exonic region then select “zoom”. After getting the exonic region of exon 1, put the cursor on the region and press right button. Then the section marked as “get DNA” was clicked. A new tab was opened. Then the chromosomal location of exons typed. The numbers of introns to be added upstream and downstream (usually not less than 50) typed. “Mask repeat” section selected to lower case. Then “get DNA” section clicked on. Desired sequence of target region will appear. The sequence copied and saved in a word file named according to gene name. After that the Primer 3plus software opened.

**Primer 3plus software:**

The sequence that was copied in UCSC browser was pasted in the section marked as “Main” at the box marked as “paste template sequence below”. Then the section marked as “General settings” clicked on. Product size was mentioned in the box marked as “Product Size Ranges”. ). Following range of Primer size was mentioned: in “Min” box-18, in “Opt” box-24 and in “Max” box-30. Following range of Primer Tm was specified in “Primer Tm” box:Min-52, opt-55 and Max-58. Maximum Tm difference (which should be 5) was mentioned. Following range of GC content was mentioned in Primer GC% box: Min-40, opt-50 and Max-60. Then

the section marked as “Pick primer” was clicked. A number of primer pair appeared. From the lists desire primer was selected after checking in IDT and custom track.

**IDT:**

Following IDT parameters were checked:

* Regions of secondary structure (Hairpin) was tried to avoid. Optimally a primer, whose 3' end hairpin with a ΔG of (-2 kcal/mol) and an internal hairpin with a ΔG of (-3 kcal/mol) were accepted.

* Runs of 4 or more of one base, or dinucleotide repeats (for example, ACCCC or ATATATAT)were tried to avoid.

* Self-dimer formation was tried to avoid. Optimally a primer, whose 3' end self-dimer with a ΔG of (-5 kcal/mol) and an internal self-dimer with a ΔG of (-6kcal/mol) were accepted.

If the IDT parameter of the primer was satisfied then the primer checked through custom track. We have used UCSC custom track to visually identify the forward and reverse primers of the exons for all four genes.

**Amplicon Preparation:**

The target regions of *BRCA1, BRCA2, ERBB2* and *TP53* genes were amplified using multiplex PCR technology. Total 13 sets of multiplex PCR were done to amplify all 52 amplicons. By implementing several trial and error methods, NeuroGen have established its own “BC plus gene panel”. To amplify each set, 15 µL of Gotaq colorless hotstart master mix, 1.2 µL (10nmol 0.6 µL of Forward primer mix + 10 nmol 0.6 µL of reverse primer mix) primer mix, 100 to 150ng of genomic DNA per reaction, and rest of nuclease free water were mixed together. The forward primer mix was prepared by taking 3µl from initial primer stock from all forward primer in each set/pool and then the rest of nuclease free water was added to make 30µl (10nm) forward primer mix. In similar way reverse primer mix was made (**see Table S5**).

**Validation:**

At first all 52 amplicons were undergone single PCR and the amplicons with a common T^m^ find out. Then they are grouped into sets. In a single set amplicons were included as such so that the sum of their product size should not exit 4000bp in case of Gotaq colorless mastermix according to the amplification capacity of this mastermix. But in set 8 and set 9 the sum of product size of amplicons are 8965 and 11910 respectively. In case of these two set gotaq long PCR mastermix is used due to the large amplification capacity of the mastermix (see Figure S1).

**Supplementary Tables:**

**Table S1:** Detail of *BRCA1* primers used

| SI NO. | FP Id. | FP Sequence | RP Id. | RP Sequence | Product Size |
| --- | --- | --- | --- | --- | --- |
|  | B1F1 | AGTAGAAGGACTGAAGAGTG | B1R1 | GAACTCATACAACCAGGACC | 324 |
|  | B1F2 | GAACATTCATATCTTACTCCCACC | B1R2 | CAGTAGTCCTACTTTGACACTT | 334 |
|  | B1F3 | AGTTCTCAAATCCTTACCCATC | B1R3 | CTGGGTTAAGTATGCAGATTACTG | 302 |
|  | B1F4 | CACTATGTAAGACAAAGGCTG | B1R4 | CCATCCAAGCCTCTTATTAAAC | 501 |
|  | B1F5 | GGCAAGATGAGTTTTCAGTG | B1R5 | CTTCTCTTTCTCTTATCCTGATGG | 583 |
|  | B1F6 | GTTAAGGAAAGTGGTGCATTG | B1R6 | CAGATTGATCTTGGGAGTG | 828 |
|  | B1F7 | GCAAGGTATTCTGTAAAGGTTC | B1R7 | GAGACTTAAAGCTAGGATAACTG | 367 |
|  | B1F8 | GTCTTAGTCATTAGGGAGATAC | B1R8 | TCTTAACAGAGACCAGAACT | 422 |
|  | B1F9 | ACCTTGATTAACACTTGAGC | B1R9 | CATTTCTGATCTCTCTGACATGAG | 472 |
|  | B1F10 | GACATATCTATCTAACCGCAC | B1 R10 | TCACTATCAGAACAAAGCAGT | 401 |
|  | B1F11 | AAGGCTCAGATACAAACACAG | B1 R11 | CCTTAACTTGTTTACAGCGATG | 320 |
|  | B1F12 | GACTGGTATATTAGTTGTGAGCAG | B1 R12 | GAACTTGTAGTTCCATACTAGGTG | 467 |
|  | B1F13 | CATACTACTGAATGCAAAGGAC | B1 R13 | CTCCAAGGTGTATGAAGTATG | 4057 |
|  | B1 F14 | CCAATTAAAAGTAAAGGGCAGG | B1 R14 | GATCTTGGTCATTTGACAGTTC | 716 |
|  | B1 F15 | GAACAGAGGAAAGAAAGAGTTG | B1 R15 | CTAGCATTGTACCTGCCAC | 729 |
|  | B1 F16 | CCATAGGGCTCATAAAATTCAC | B1 R16 | CAAATCACACATATCCCACAC | 572 |
|  | B1 F17 | CTCAATAAAGAGATGTTGCCAG | B1 R17 | AGGTTGATAATCACTTGCTGAG | 1225 |
|  | B1 F18 | GCAGATGTCCCATAAAACTTTC | B1 R18 | CTTGTAATTCACCTGCCATTAC | 397 |
|  | B1 F19 | TATACTCTCTGAGAAAGAATGAAATGGAG | B1 R19 | CATGAGTTGATGGTTGTTGG | 749 |
|  | B1 F20 | CCCTAGTATGTAAGGTCAATTCTG | B1 R20 | GACGTTGTCATTAGTTCTTTGG | 328 |

**Table S2:** Detail of *BRCA2* primers used

| SI No. | FP Id. | FP Sequence | RP Id. | RP Sequence | Product Size |
| --- | --- | --- | --- | --- | --- |
|  | B2F1 | GTTTTGGGGAAGTGTTTTACAG | B2R1 | GAACTCATACAACCAGGACC | 637 |
|  | B2F2 | CTGGTTAAAACTAAGGTGGGAT | B2R2 | TATCAAAGGAGGGATGAAAGAG | 518 |
|  | B2F3 | ATTCTCATTCCCAGTATAGAGGAG | B2R3 | CTGGGTTAAGTATGCAGATTACTG | 1700 |
|  | B2F4 | TCACTGTGTTGATTGACCTT | B2R4 | CTTCTGAAATCCAGTAGTGTTCTG | 346 |
|  | B2F5 | CGATCTGATAACCAAGACAACTAC | B2R5 | CCTGTAGTTCAACTAAACAGAG | 521 |
|  | B2F6 | CTGTTTCTATGAGAAAGGTTGTGAG | B2R6 | CAGATTGATCTTGGGAGTG | 1266 |
|  | B2F7 | GCCCAAACACTACCTTTTTAAC | B1R7 | GAGACTTAAAGCTAGGATAACTG | 5101 |
|  | B2F8 | GGATTTATCCTGTTTAGACCCT | B2R8 | AACTGTCAACTTTCGGAAGATC | 657 |
|  | B2F9 | GAGCATCTGTTACATTCACTGA | B2R9 | CATTTCTGATCTCTCTGACATGAG | 409 |
|  | B2F10 | GTATCACCATGTAGCAAATGAG | B2R10 | TCACTATCAGAACAAAGCAGT | 611 |
|  | B2F11 | GAGAAAAGGAGAGCATGTAAAC | B2R11 | CCTTAACTTGTTTACAGCGATG | 846 |
|  | B2F12 | AGCAGACTGTGGAATGTATG | B2R12 | GAACTTGTAGTTCCATACTAGGTG | 448 |
|  | B2F13 | TCAGTATCATCCTATGTGG | B2R13 | CTCCAAGGTGTATGAAGTATG | 1264 |
|  | B2F14 | GGCAGTTCTAGAAGAATGAAAACTC | B2R14 | GATCTTGGTCATTTGACAGTTC | 1253 |
|  | B2F15 | GAACAGAGGAAAGAAAGAGTTG | B2R15 | CTAGCATTGTACCTGCCAC | 802 |
|  | B2F16 | CATTAACCACACCCTTAAGATG | B2R16 | CATGGTGTCAAGTTTCTCTTC | 1042 |
|  | B2F17 | GGTCATTTTGGAAAACCTGAG | B2R17 | AGGTTGATAATCACTTGCTGAG | 483 |
|  | B2F18 | GGTCCAAACTTTTCATTTCTGC | B2R18 | CTTGTAATTCACCTGCCATTAC | 490 |
|  | B2F19 | TAACCTATTAGGAGTTAGGGGAG | B2R19 | TTGACTCAGTCATAACAGCTC | 931 |

**Table S3:** Detail of *ERBB2* primers used

| SI No. | FP Id | FP Sequence | RP Id | RP Sequence | Product Size |
| --- | --- | --- | --- | --- | --- |
|  | E2F1 | AAAAGTCCTTTCGATGTGACTG | E2R1 | CATTCTTATACTTCCTCAAGCAGC | 574 |
|  | E2F2 | CTCTCTTGGGGGATTTTTACC | E2R2 | CACCCACCTGTAAACAGAGG | 591 |
|  | E2F3 | CCTTTGCTTTCACTGATGAAG | E2R3 | CTCTGAATAACCAAGAGAAGG | 549 |
|  | E2F4 | CTCTTTTAGAAGGCAGGAGG | E2R4 | CCACCAAAATGAGAAAACTGTG | 1288 |
|  | E2F5 | GGTAGGGCATTTAAGTATTGGTTG | E2 R5 | GATCCTCAGGACTCTGTCTG | 695 |
|  | E2F6 | CTGCTGACTCCTCTCCTGAC | E2 R6 | CTCCAGGGATATTTTACCAGATAG | 1464 |
|  | E2F7 | GAAGAGCAAGGGTGTTTGTC | E1 R7 | GTTGGTCCCCTTTTATAGTAAGAG | 394 |
|  | E2F8 | CGGTGTTAGAACATGGAAAAAC | E2 R8 | GAAGGGCAATGAAGGGTACATC | 549 |
|  | E2F9 | GCAGGTTTTAGAGTAGGAGAG | E2R9 | GGA AGG TTC CTT AGG ACA GG | 5025 |

**Table S4:** Detail of *TP53* primers used

| SI No. | FP Id. | FP Sequence | RP Id | RP Sequence | Product Size |
| --- | --- | --- | --- | --- | --- |
|  | TF1 | ATTGAAGTCTCATGGAAGCC | TR1 | AGGGTTGGAAGTGTCTCATG | 715 |
|  | TF2 | GCTATGATGTTCCTTAGATTAGGTG | TR2 | AAAAAGAAAAGCTCCTGAGGTG | 2240 |
|  | TF3 | GAATGTGGTTATAGGATTCAACCG | TR3 | TGTATATACTTACTTCTCCCCCTC | 323 |
|  | TF4 | CAAAGACCCAAAACCCAAAATGGC | TR4 | TACACACTAATACTCTGAGGTG | 341 |

In total the BC Plus Gene panel consists of 52 sets of primer.

**Table S5:** Detail of multiplex PCR

| Set Id | Primer Id | Amplicon Size | PCR condition | PCR ReactionVolume (µL) |
| --- | --- | --- | --- | --- |
|  |  |  |  |  |
| Set 1 | B1(F6+R6) | 828 | 95-2min,[ 95-30s, **50**-30s, 72-**50s**] 30 cycle, 72-5min,4-hold | 30 |
|  | B2(F10+R10) | 611 |  |  |
|  | E2(F8+R8) | 549 |  |  |
|  | E2(F7+R7) | 394 |  |  |
| Set 2 | B2(F15+R15) | 802 | 95-2min,[ 95-30s, **52**-30s, 72-**50s**] 35 cycle, 72-5min,4-hold | 30 |
|  | B2(F1+R1) | 637 |  |  |
|  | B1(F5+R5) | 583 |  |  |
|  | B2(F5+R5) | 521 |  |  |
|  | B2(F12+R12) | 448 |  |  |
|  | B1(F2+R2) | 334 |  |  |
| Set 3 | B1(F14+R14) | 716 | 95-2min,[ 95-30s, **52**-30s, 72-**50s**] 35 cycle, 72-5min,4-hold | 30 |
|  | B1(F16+F16) | 572 |  |  |
|  | B2(F2+R2) | 518 |  |  |
|  | B1(F12+R12) | 467 |  |  |
|  | B2(F9+R9) | 409 |  |  |
|  | T(F4+R4) | 341 |  |  |
| Set 4 | B2(F6+R6) | 1266 | 95-2min,[ 95-30s, **52**-30s, 72-**1.20s**] 35 cycle, 72-5min,4-hold | 30 |
|  | B1(F19+R19) | 749 |  |  |
|  | E2(F3+R3) | 542 |  |  |
|  | B1(F9+R9) | 472 |  |  |
|  | B1(F10+R10) | 401 |  |  |
|  | B1(F1+R1) | 324 |  |  |
| Set 5 | B2(F16+R16) | 1042 | 95-2min,[ 95-30s, **52**-30s, 72-**1.0m**] 35 cycle, 72-5min,4-hold | 30 |
|  | T(F1+R1) | 715 |  |  |
|  | B1(F7+R7) | 367 |  |  |
|  | T(F3+R3) | 323 |  |  |
| Set 6 | B2(F13+R13) | 1264 | 95-2min,[ 95-30s, **52**-30s, 72-**1.20s**] 35 cycle, 72-5min,4-hold | 30 |
|  | B1(F15+R15) | 729 |  |  |
|  | E2(F1+R1) | 574 |  |  |
|  | B2(F17+R17) | 483 |  |  |
|  | B2(F4+R4) | 346 |  |  |
| Set 7 | B2(F14+R14) | 1263 | 95-2min,[ 95-30s,**48**-30s, 72-**1.20s**] 35 cycle, 72-5min,4-hold, | 30 |
|  | B2(F19+R19) | 931 |  |  |
| Set 8 | E2(F9+R9) | 5025 | Cycle: 95-2min,[ 95-30s, **51**-30s,72-**5min**] 35cycle, 72-5min,4-hold | 30 |
|  | T(F2+R2) | 2240 |  |  |
|  | B2(F3+R3) | 1700 |  |  |
| Set 9 | B2(F7+R7) | 5101 | 95-2min,[ 95-30s,**48**-30s, 72-**5min**] 32 cycle, 72-5min,4-hold | 30 |
|  | B1(F13+R13) | 4057 |  |  |
| Set 10 | E2(F2+R2) | 591 | 95-2min,[ 95-30s, **52**-30s, 72-**35**s] 35 cycle, 72-5min,4-hold | 30 |
|  | B1(F4+R4) | 501 |  |  |
|  | B1(F18+R18) | 397 |  |  |
|  | B1(F11+R11) | 320 |  |  |
| Set 11 | E2(F6+R6) | 1464 | 95-2min,[ 95-30s, 57-30s, 72-1.40s] 35 cycle, 72-5min,4-hold | 30 |
|  | E2(F4+R4) | 1288 |  |  |
|  | E2(F5+R5) | 695 |  |  |
|  | B1(F3+R3) | 302 |  |  |
| Set 12 | B1(F17+R17) | 1225 | 95-2min,[ 95-30s, **51**-30s, 72-**1.20s**] 35 cycle, 72-5min,4-hold | 30 |
|  | B2(F11+R17) | 846 |  |  |
|  | B2(F18+R18) | 390 |  |  |
| Set 13 | B2(F8+R8) | 657 | 95-2min,[ 95-30s, **48**-30s, 72-**40s**] 35 cycle, 72-5min,4-hold | 30 |
|  | B1(F8+R8) | 422 |  |  |

**Table S6:** Detail of primers for Sanger sequencing

| **Identified mutations** | **Coordinate** | **Primer sequence** |
| --- | --- | --- |
| TP53_F_7578490_c.322dupG | chr17:7578226-7578245 | TCATCCAAATACTCCACACG |
| TP53_R_7578490_c.322dupG | chr17:7578600-7578621 | CTTCCAGTTGCTTTATCTGTTC |
| BRCA2_F_32899247_c.351_352del | Chr13:32899091-32899114 | CAACTCCCTATACATTCTCATTCC |
| BRCA2_R_32899247_c.351_352del | Chr13:32899523-32899544 | GAAAACCAGCCAATTCAACATC |
| BRCA2_F_32906916-32906923 _AAAGAAAG_c.1301_1308del | Chr13:32906464-32906483 | TGGAAAGTCAATGCCAAATG |
| BRCA2_R_32906916-32906923 _AAAGAAAG_c.1301_1308del | Chr13:32907062-32907081 | GATATTGCCTGCTTTACTGC |
| BRCA1_F_41222983_c.T5011 T>C | chr17:41222907-41222928 | GTCTTAGTCATTAGGGAGATAC |
| BRCA1_R_41222983_c.5011 T>C | chr17:41223309-41223328 | TCTTAACAGAGACCAGAACT |
| ERBB2_R_37880988_c.2272 G>C | Chr13:37881304-37881323 | CATCCTCCAGGTAGCTCATC |
| BRCA2_R_32914943_c.6451 G>A | chr13:32915389-32915410 | TCAGTAGACTTGGTATGCTAAC |

**Table S7:** Detail of the history of study cases who have mutations

| **SI No.** | **Sample Type** | **Age range (years)** | **Clinical information** | **Stage of cancer** | **Grading** | **Gene Name** | **Nucleotide change** | **Mutation Classification** |
| --- | --- | --- | --- | --- | --- | --- | --- | --- |
| 1 | Blood | 32-62 | BC | II | I | *BRCA2* | c.1792A>G | VUS |
| 2 | Blood |  | BC | I | II | *BRCA1* | c.5011T>C^*^ | Pathogenic |
| 3 | Blood |  | BC | II | III | *ERBB2* | c.2272G>C^*^ | VUS |
| 4 | Blood |  | BC | II | II | *BRCA2* | c.351_352delTC^*^ | Pathogenic |
| 5 | Blood |  | BC | III | III | *BRCA1* | c.2286 A>T | VUS |
| 6 | Blood |  | BC | II | II | *BRCA1* | c.3995C>A | VUS |
| 7 | Blood |  | BC | III | II | *BRCA2* | c.1301_1308del AAAGAAAG^*^ | Pathogenic |
| 8 | Blood |  | BC | II | II | *BRCA1* | c.1058G>A | Pathogenic |
| 9 | Blood |  | No symptoms | N/A | N/A | *BRCA2* | c.7722G>A | Pathogenic |
| 10 | Blood |  | Breast pain and swelling | N/A | N/A | *BRCA2* | c.1114A>C | VUS |
| 11 | Blood |  | Breast lump | N/A | N/A | *BRCA2* | c.6451G>A^*^ | Pathogenic |
| 12 | Tissue |  | BC | III | missing | *TP53* | c.733G>A | Pathogenic |
| 13 | Tissue |  | BC | III | missing | *TP53* | c.322dupG^*^ | Pathogenic |
|  |  |  |  |  |  | *BRCA1* | c.116G>A | Pathogenic |
| 14 | Tissue |  | BC | missing | missing | *BRCA2* | c. 2459A>G | VUS |

**Note: BC means Breast Cancer**
